# Pediatric psychiatric emergency rooms during COVID-19: a multi-center study

**DOI:** 10.1186/s12888-022-04371-7

**Published:** 2022-12-27

**Authors:** Galit Erez, Sol Yakubovich, Hadar Sadeh, Gal Shoval, Gila Schoen, Gal Meiri, Nimrod Hertz-Palmor, Tali Bretler, Yael Barzilai, Mariela Mosheva, Doron Gothelf, Yuval Bloch

**Affiliations:** 1 Shalvata Mental Health Center and Tel Aviv University, Hod Hasharon, Israel; 2 Shalvata Mental Health Center, Hod Hasharon, Israel; 3 Soroka Medical Center and Ben-Gurion University of the Negev, Be’er Sheva, Israel; 4 Geha Mental Health Center and Tel Aviv University, Petah Tikva, Israel; 5 Sheba Medical Center and Tel Aviv University, Ramat Gan, Israel; 6 Ziv Medical Center (Safed) and Bar-Ilan University, Safed, Israel

**Keywords:** COVID-19, Psychiatric emergency room, Children & adolescents, Stress-related, Severe mental disorder

## Abstract

**Background:**

The COVID-19 (SARS-CoV-2) pandemic has been a major stressor for the mental health and well-being of children and adolescents. Surveys and reports from hotlines indicate a significant rise in mental health problems. As the psychiatric emergency room (ER) is a first-line free-of-charge facility for psychiatric emergencies, we expected to see a significant increase in visits, specifically of new patients suffering from anxiety, depression, or stress-related disorders.

**Methods:**

Data from two psychiatric hospital ERs and one general hospital were included. All visits of children and adolescents from the computerized files between March and December of 2019 were analyzed anonymously and compared to the same months in 2020, using multilevel linear modeling.

**Results:**

There was a significant decline in the total number of visits (*p* = .017), specifically among those diagnosed as suffering from stress-related, anxiety, and mood disorder groups (*p* = .017), and an incline in the proportion of visits of severe mental disorders (*p* = .029).

**Discussion:**

The limited use of child and adolescent psychiatric emergency facilities during the pandemic highlights the importance of tele-psychiatry as part of emergency services. It also suggests the importance of the timeline of the emergence of clinically relevant new psychiatric diagnoses related to the pandemic. Future studies are needed to establish the long-term effects of the pandemic and the expeditious use of tele-psychiatry.

**Supplementary Information:**

The online version contains supplementary material available at 10.1186/s12888-022-04371-7.

## Background

The COVID-19 pandemic has been a major stressor for the mental health and well-being of children and adolescents, resulting in an accumulation of worldwide reports regarding the severe and multifaceted consequences on the mental health of this cohort. Many of these studies have been based on community surveys and thus reflect a rise in stress, although not necessarily an incline in psychopathology [1–3]. Lay publication reports from hotline services which do not usually report characteristics such as age, due to the anonymity of the calls and community volunteer organizations present an increase in anxiety, depression, deliberate self-harm and suicidal thoughts, [4–7].

A routine suicide screening in a large pediatric emergency department identified a rise in both suicide risk and attempts [8]. On the other hand, a study that systematically assessed the entire child and adolescent population of Japan based on a central registry found no rise in rates of completed suicides during the pandemic [9], although a later study covering the second half of 2020 found a rise in the suicide rate among the same population (Japan), mainly among young adults [10]. Still, a recent study that included 21 countries found no increase, and in some states, a decrease in the comparative suicide rate [11].

Beyond suicidal risk, surveys and studies support a rise in anxiety depression and stress related symptoms in children and adolescents [2, 12]. It is important to be more specific relating to different risk factors that typify some youngsters who are in increased mental health risk during the pandemic. A recent non-systematic review attempted at bringing forth a more general understanding of the child and adolescent mental health implications of the Covid-19 pandemic [12]. They stress previous mental health problems as a probable risk factor (among other risk factors) for additional mental health pathology.

In line with this, a behavioral deterioration during the pandemic was reported in children and adolescents with severe developmental disorders such as autism spectrum disorder [13]. Other studies have recognized the detrimental effect of other less debilitating developmental difficulties such as ADHD on mental health of adolescents during the Covid-19 pandemic [14].

Since visits to the emergency room (ER) are a manifestation of acute need and are less likely to be postponed during the pandemic some studies focused on emergency room visits as a way to evaluate severe mental health consequences.

ER referrals of pediatric patients to a tertiary center in Oregon due to suicide attempts during the first two months of the pandemic and lockdown showed a sharp decline [15]. A study that included all ER visits for psychiatric pediatric consultations in a single ER from New Haven, during the first two months of the pandemic found a reduction of more than 60% in visits during the pandemic [16].

The current study aimed to evaluate clinically relevant acute mental states among children and adolescents from different centers in Israel during the pandemic. As such, we focused on emergency room psychiatric referrals from three centers in Israel, not just at the beginning of the pandemic (i.e., during the initial lockdown), but from March to December 2020, thereby covering the fluctuations in the pandemic waves. This period was unique because it was before vaccines were available. There were three general lockdowns during the study period. Lockdowns dates were 25/3/2020–4/5/2020; 18/09/2020–17/10/2020; 17/12/2020–07/02/2021. Our focus was neither inclusive of all the stress and deterioration in mental health in the general child-adolescent population, nor limited to “tip of the iceberg” suicidal behavior or completed suicide. Rather, we used all ER visits. ER visits are typified as a perceived acute psychiatric emergency by family or by the minor. Israel is unique because of the divergent cultural and ethnic background (Jews and Arabs, conservatives, ultrareligious and secular, etc.), all inhibiting a small geographic area. Thus, the children and adolescents studied although divergent, encountered similar challenges relating to the pandemic. Including school closure, the lockdowns, and precautions such as seclusions, use of face masks, and social distancing.

We hypothesized that in our capturing of a prolonged period during the pandemic, there would be a rise in pediatric psychiatric ER visits. Given the overall stressful situation, we expected a rise in visits of patients suffering from stress-related disorders, including the presentation of anxiety and depression. We expected to find many more patients with no previous psychiatric background compared to the pre pandemic period.

## Method

### Sample

It is important to note that the Israeli population is heterogeneous in ethno-cultural origin, religion, and type of residency and thus probably allows for better generalization to different populations. During the pandemic there were more cases of infection among the Muslim minority and the Ultraorthodox Jewish community in comparison to the general Israeli population (possibly at least partially due to less awareness of the dangers of the pandemic and more suspiciousness about regulations set by the government) [17].

Three ERs were included in the study, two of which are located in psychiatric centers.The Shalvata Mental Health Center serves all age groups. It covers a population of approximately 500,000 inhabitants in the center of Israel. The catchment area includes a heterogeneous population of both Jews and Muslims. The Geha Mental Health Center serves all age groups. It covers a population of approximately 800,000 inhabitants and covers a comparatively large population of Ultraorthodox Jews. Soroka Medical Center (a general hospital that covers all age groups), is in the southern part of Israel, serves a population of approximately one million inhabitants. Most of this population is considered part of Israel’s “periphery” and includes both rural and non-rural habitants and a large population of Bedouins (a Muslim population with a culture and tradition of its own).

In Israel there is a continuous growth of the population and a yearly incline in the use of pediatric psychiatric services in general, and in psychiatric ER visits specifically. For example, at Shalvata the number of yearly visits to the psychiatric ER of pediatric patients grew from 827 visits in 2017, to 910 visits in 2018, to 1,040 visits in 2019.

The current study focused on all children and adolescents who arrived in the three above-mentioned ERs from March to December 2020 (the first patient to be diagnosed with COVID-19 in Israel was on February 21^st^, 2020) in need of emergency psychiatric evaluation. A comparison was made with ER visits during the same months in 2019.

Children were defined as under the age of 12, including 12, and adolescents 13–18 including 18.For the two psychiatric hospitals all child and adolescent visits were included, for the general hospital all child and adolescent visits who were discharged after psychiatric consultation and received a psychiatric diagnosis (code f in the ICD-10) were included.

A total of 3,224 patients arrived within this comparison-based timeline to all three ER departments: 1,735 of them arrived in 2019 and the remaining 1,489 in 2020 (Table 1).

**Table 1:** Demographic variables and diagnoses of patients

	**Total (*n* = 3,224) 100%**	**March—December 2019 (*n* = 1,735) 53.81%**	**March – December 2020 (*n* = 1,489) 46.18%**
Male (%)	45.43%	46.93%	43.94%
Age (mean ± SD)	13.78 ± 3.01	13.79 ± 2. 99	13.77 ± 3.03
Jewish (%)	69.38%	66.88%	68.89%
Urban residence (%)	56.02%	56.37%	55.67%
Born in Israel (%)	86.93%	87.61%	86.26%
Diagnoses			
Stress-related, anxiety, and mood disorders	25.15% (*n* = 811)	27.99% (*n* = 485)	23.37 (*n* = 347)
ADHD, conduct, and learning disabilities	23 .52% (*n* = 758)	23.55% (*n* = 409)	23.50% (*n* = 350)
Severe mental disorders	46.27% (*n* = 1492)	45.43% (*n* = 789)	47.21% (*n* = 703)
Other diagnoses^b^	5.06% (*n* = 163)	3.03% (*n* = 52)	5.97% (*n* = 89)
Psychiatric history			
Yes^a^	37.5%	32.5%	43.5%

^a^ For Shalvata and Soroka only

^b^ Other diagnoses' are too small to consideration

### Measures

For descriptive statistics of the sample, demographic variables were examined: sex, age, religion, type of residence, and country of birth. Religious affiliation was classified as Jewish or Muslim. Type of residence was categorized into urban versus rural. Country of birth was dichotomously divided into “born in Israel” vs. “born abroad.”

#### Education system

During the pandemic, the special education system continued primarily with frontal teaching, whereas the regular education system was primarily characterized by distance learning and significantly fewer formal encounters with peers, teachers, and school counselors. We thus divided the ER visits based on whether the child or adolescent was in the special education system. The education system was reported in only one of the centers (Shalvata).

#### Diagnoses

The diagnosis of each patient in accordance with the international statistical classification of diseases and related health problems (ICD-10) was based on the clinical diagnosis given by the psychiatrist in the ER and documented in the computerized files. We gathered diagnoses into three groups: 1) anxiety, mood, and stress-related disorders; 2) ADHD, conduct, and learning disability disorders; and 3) severe mental illnesses (other). The full list of diagnoses classified into each category is presented in the Additional file 1. Other diagnoses were not included in the analyses due to very low frequencies. As a result of comorbidities, many patients may have had more than one diagnosis. Thus, the prevalence of each diagnostic group was calculated relative to the total number of diagnoses (Table 1).

#### Psychiatric history

Psychiatric history was a dichotomous variable based on whether the patient had been treated in the outpatient clinic in the past two years. Assessing this variable was possible only at Shalvata and the Soroka Mental Health Center.

#### Outcome of the ER visit

For the two psychiatric ERs (Shalvata and Geha), we compared dichotomously whether the patient had been hospitalized or discharged.

### Procedure

Data was collected from the electronic files of all three centers. Measurements were extracted monthly from January 2019 to December 2020. As mentioned, given that the first case of COVID-19 in Israel was diagnosed on February 21^th^, 2020, the months of January and February of both years (i.e., 2019 and 2020) were removed from the analyses.

### Data analysis

Multilevel linear modeling (mixed-effects linear models) was applied to assess changes in ER visits, prevalence of diagnoses, and rates of hospitalization between 2019 and 2020. To account for the heterogeneity between the centers and time-dependent fluctuations across different months, random effects included the medical center and month of the year from which data were extracted. In the diagnoses model (Model I), we also included the interaction between each medical center and diagnosis prevalence as a random effect, to overcome baseline differences in prevalence between the centers in ER visits due to psychiatric morbidity. Analyses were conducted using the lmerTest package in R [18].

We used non-parametric chi-square tests for independence to analyze partial data which were not available from all three centers (i.e., education system and psychiatric history). Effect sizes are presented via change in parameter prevalence between 2019 and 2020.

## Results

### Visits to the ER during the pandemic

**Fig. 1:**
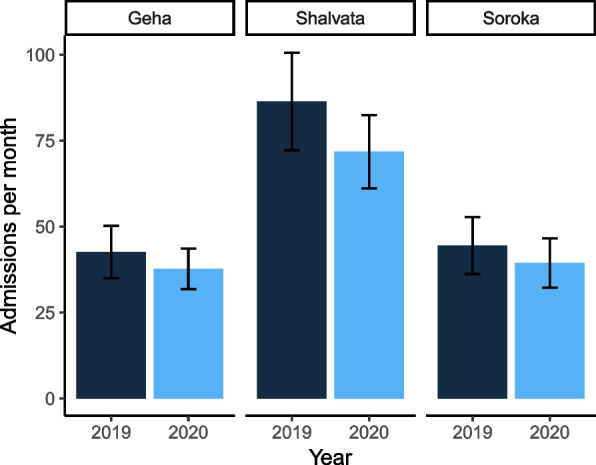
Visits to the ER before and during the pandemic

The rate of total pediatric acute psychiatric ER visits decreased significantly during 2020. In 2019, the mean was 57.83 ± 26.23 monthly visits to the ER. In 2020, the mean was 49.63 ± 20.40 monthly visits (*Unstandardized B (47)* = -8.20, *95% CI* = -13.25, -3.14, *p* = .002) (Fig. 1). In 2019 there were 864 visits in Shalvata, 426 visits in Geha, and 445 visits in Soroka. In 2020, there were 718 visits, 377 visits and 394 visits, accordingly.

### Diagnoses of patients who visited the ER

The prevalence and proportion of stress-related, anxiety, and mood disorders decreased significantly from 2019 (27.9% ± 16.8% of all visits) to 2020 (22.3% ± 14.0%, *B (168)* = -0.06, *95% CI* = -0.10, -0.01, *p* = 0.017).

The prevalence of severe psychiatric disorders did not change significantly but the proportion of these disorders from the overall referrals increased significantly across centers (from 45.4% ± 12.4% in 2019, to 47.2 ± 13.5% in 2020, *B (168)* = 0.07, *95% CI* = 0.01, 0.14, *p* = 0.029), but was inconsistent between centers.

**Fig. 2:**
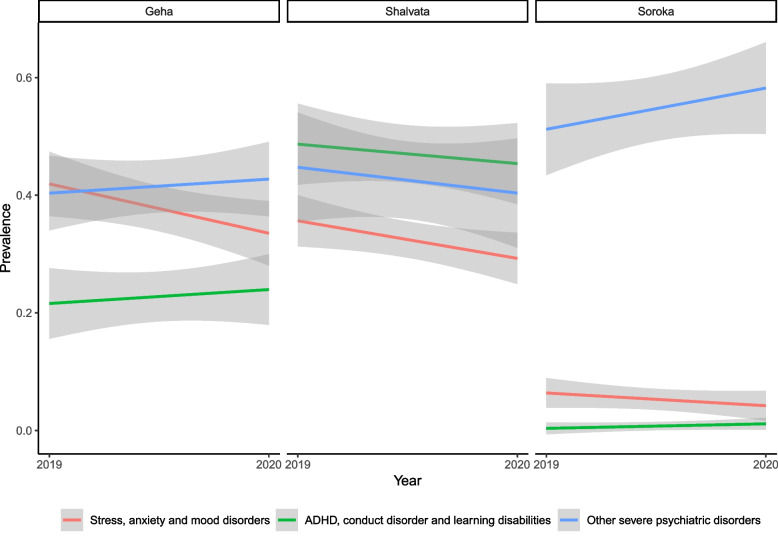
Diagnosis of patients visiting the ER before and during the pandemic

The prevalence and proportion of ADHD, conduct disorder, and learning disabilities remained unchanged (Fig. 2).

### Outcome (i.e., psychiatric hospitalization vs. discharge)

The prevalence of visits that resulted in patient hospitalization did not change significantly between 2019 (27.9% ± 9.6%) and 2020 (26.5% ± 10.4%).

### Education system

In 2019, 33.33% of the ER visits were from patients studying in the special education system, and in 2020, 36.21% ER visits were from this same cohort. A chi-square test was used, with special education (yes or no) and year (2019 or 2020) as variables. No correlation was found, χ^2^_(1)_ = 0.06, *p* = 0.81.

### Psychiatric history

A chi-square test was used, with psychiatric history (yes or no) and year (2019 or 2020) as variables. A significant correlation was found, χ^2^_(1)_ = 31.35, *p* = 0.00, with a higher percentage of patients who had a previous history at the clinic visiting in 2020 (in 2019, 32.5% were patients with such a history, compared to 43.5% in 2020).

### Multilevel linear modeling (mixed-effects linear models)

The multilevel models substantiated a decline in ER visits during the pandemic specifically for patients diagnosed with stress, anxiety, and mood-related disorders. During the pandemic there was a relative incline in the visits of patients with severe psychiatric morbidity and those who had been treated in the past (psychiatric history recorded in a single center) (Table 2).

**Table 2:** Multilevel models comparing trends in 2020 (as fixed factor) compared with equivalent month in 2019 (reference event)

Multi center mixed-effects linear models				
**Model**	**Outcome**	**B (95% CI)**		**p**
**1**	Visits to the ER^a^	-8.20 (-13.25, -3.14)		**.002**
**2**	% Stress related, anxiety and mood disorders^b^	-0.06 (-0.10, -0.01)		**.017**
	% ADHD, conduct and learning disabilities^b^	0.06 (-0.01, 0.12)		.09
	% Other severe psychiatric morbidity^b^	0.07 (0.01, 0.14)		**.029**
**3**	% Hospitalization^c^	-0.01 (-0.05, 0.02)		.39
**Single center non-parametric tests**				
**Test**	**Outcome**	**Δ Prevalence (2020 minus 2019)**	**Χ**^**2**^** (df = 2)**	**p**
**1**	Psychiatric history^e^	+ 15.3%	26.79	** < .001**
**2**	Special education^d^	+ 2.88%	0.06	.81

^a^ Outcome measure: Average number of visits per month

^b^ Outcome measure: Prevalence of psychiatric disorders among overall monthly visits to the ER (0 ≤ % ≤ 1); Modeled as the interaction between each diagnosis and prevalence in 2020

^c^ Outcome measure: Prevalence of visits which resulted in hospitalization (0 ≤ % ≤ 1)

^d^ Outcome measure: Prevalence of patients studying in special education institutes (0 ≤ % ≤ 1)

^e^ Outcome measure: Prevalence of patients previously known to the clinic (0 ≤ % ≤ 1)

## Discussion

The current study presents a decline in visits of children and adolescents to psychiatric ERs during the pandemic. This decline is most prominent in visits of patients diagnosed with stress-related, anxiety, and mood disorders as discussed below. Although this finding-decline in ER visits during the pandemic is counterintuitive, given the rise of distress during this period in children and adolescents [8, 19] it is in accord with current single center studies of psychiatric ERs [15, 16, 20]. Large-scale studies of general pediatric emergency room visits have found an even more pronounced decline during this period in ER pediatric visits for different disorders [21–23].

It is important to arrive at a better understanding of this decline in psychiatric ER visits among children and adolescents. Such an understanding is of value both from an immediate clinical perspective, as the pandemic continues worldwide, and may also contribute to a better understanding of the trajectory from a complex stressful situation (the pandemic) to emotional and behavioral pediatric emergencies as presented by ER visits.

A possible explanation for this study’s findings would be that the decline in ER visits relates to difficulties in access to emergency psychiatric care and not to a smaller incidence of psychiatric emergencies.

It has been suggested that the fear of arriving at hospitals and becoming infected during the pandemic is a central reason for the decline in such visits [23]. The current multi-center study included two psychiatric ERs outside the premises of a general hospital, and so fear of covid is not likely as the only explanation for what occurred in these facilities, and yet the decline in referrals was the same in all three ERs. In addition, this longer time-period study (10 months), which included prolonged periods during which there were less restrictions on transportation, and less fear of being infected by reaching medical facilities, makes difficulties in access to the ER less likely as a sole explanation to the decline in ER visits. Still as during the first ten months of the pandemic, there was general fear of infection, and immunizations were not present yet, we consider this fear a probable significant reason for the observed decline.

In the Israeli psychiatric system, psychiatric ER visits are free of charge; it is therefore not likely that the economic uncertainty typifying the lives of many families during the pandemic was a central reason for the decline in pediatric visits to the psychiatric ER.

Another suggested explanation is that during the pandemic, only individuals suffering from more severe emergencies would arrive. This explanation is in accord with the relative rise in the proportion of referrals of patients with severe mental health disorders, but important to stress that even with these diagnoses there was a non-significant decline in the numerical number of visits. However, if this had been the case, then a larger proportion of the ER visits would have resulted in hospitalization rather than in discharge. The current findings are in accord with a study that covered most of the psychiatric hospital beds for adolescents in Israel and showed a decline in 2020, probably at least partly related to inpatient services and mental health policies [24]. In the present study, there was no rise in the proportion of hospitalizations from ER visits; as such, the severity of the emergency seems not to have been the central reason for the decline.in ER referrals during the pandemic.

Regarding ER diagnosis, the significant decline was found in the stress-related, anxiety, and depression diagnostic group, in the other diagnostic groups, there was a numerical decline that did not reach significance. A few of the previous studies evaluated diagnosis. In the large-scale study relating to all pediatric ER visits during the pandemic in 27 hospitals in the USA [23] there was a 38% decline in visits of patients diagnosed with depression, a 37.8% decline in visits of patients with trauma and stress-related disorders, and a 16% decline in visits of patients with schizophrenia spectrum and other psychotic disorders (this last finding perhaps being similar to the present finding of an eleven percent decline that did not reach significance in the diagnostic group of “severe mental disorders”). It is important to note that in this large-scale study just mentioned, there was only a 4% decline in visits of patients with suicidal ideation, suicidal attempt, or intentional self-harm. This finding possibly reflects the experience of general medical facilities, which provide immediate general medical help, beyond the psychiatric assessment and intervention.

In the present study, the patients who came to the ER during the pandemic were more likely to have had a past psychiatric history (i.e., they had visited the outpatient clinics previously). This finding can be in accord with the findings from the general pediatric ER, where there was an increase in the proportion of visits of patients with chronic conditions [23] In the context of psychiatric care there are a few probable explanations for this comparative incline in patients already known to the facility. In times of stress, such as the pandemic, they are probably more aware of the importance and possible use of emergency psychiatric care. In addition, when combining this finding with the diagnostic finding, they probably suffer as a group from more severe psychopathology.

In addition, a larger segment of our patients with a psychiatric history receives special support in school or are part of the special education system. During the pandemic, regular schools but not special education institutions were closed, and regular schools were functioning based on tele-education.

The effect of school closure on mental health is complex. On the one hand, the lack of routine and meeting peers in school intensifies stress and loneliness [25]. On the other hand, as pupils did not attend school physically, and many parents spent much more time at home, it is likely that the more functional and resilient families could support their kids, resulting perhaps in a reduction of various negative school-related phenomena such as bullying, ostracizing, and shaming, with a subsequent reduction of some mental stress for children and adolescents [15]. However, staying at home can also be a mental health risk factor, especially given the reported rise in domestic violence during the pandemic and lockdowns [26, 27]. In the current study, probably due to different and personal effects of the school system on psychiatric emergencies, there was no change in the proportion of referrals based on regular vs. special education.

In terms of limitations, the current study is a retrospective chart review of three centers, and thus has all the limitations typical of these types of studies. Specifically, the evaluations were clinical and not structured, and our analysis depends on the information provided in files relating to visits per month, and not to specific patients (in order to comply with regulations). It is possible that some of the psychiatric emergencies were handled in outpatient clinics or in private practices, and thus, although we used three different centers serving a heterogeneous and reasonably sized population, some information may be missing. In addition, this study covered a 10-month period of the pandemic; it is possible that some of the effects might emerge in the aftermath of the pandemic.

## Conclusions

Our findings show a decline in the use of the psychiatric ER by children and adolescents during the first year of the pandemic. This is especially evident for those who suffer from stress related disorders, anxiety or depression, and who are not acquainted to our facilities One conclusion that can be drawn from the current study is the need to develop better and more integrated emergency tele-psychiatric services as part of psychiatric emergency services [5, 6].There are guidelines for the proper use of tele-psychiatry [28], which were updated prior to the pandemic [29], and reports that have substantiated the importance of tele-psychiatry in the emergency setting [30]. The necessity of using tele-psychiatry during the pandemic advanced its use considerably [31]. Nonetheless, it seems that the option of using emergency tele-psychiatric evaluation for children and adolescents, especially “new” patients, was not exploited. In our centers, there was no virtual alternative to the ER. In a recent Canadian study on a virtual pediatric ER, only 15 of 1,036 evaluations were for “mental problems” [32]. In Israel, some of the child and adolescent psychiatric emergencies were evaluated by hotlines, which reported an incline in calls during the pandemic [33]. These hotlines serve an extremely important role in terms of providing support and making referrals to mental health therapies, but they are not a substitute for psychiatric evaluation. We believe that in psychiatric emergencies if distant evaluation is needed it should be performed using video tele-psychiatry by trained psychiatrists. Thus, we consider the results of this study to be a call for further developments in the study and incorporation of emergency tele-psychiatry for children and adolescents.

## Supplementary Information

**Additional file 1.**

## Data Availability

The datasets analyzed during the current study are available from the corresponding author on reasonable request.

## Abbreviations

COVID-19: SARS-CoV-2
ER: Emergency room

## References

[1] Patrick SW, Henkhaus LE, Zickafoose JS, Lovell K, Halvorson A, Loch S (2020). Well-being of parents and children during the COVID-19 pandemic: a national survey. Pediatrics..

[2] Duan L, Shao X, Wang Y, Huang Y, Miao J, Yang X (2020). An investigation of mental health status of children and adolescents in china during the outbreak of COVID-19. J Affect Disord..

[3] Esposito S, Giannitto N, Squarcia A, Neglia C, Argentiero A, Minichetti P (2021). Development of psychological problems among adolescents during school closures because of the COVID-19 lockdown phase in Italy: a cross-sectional survey. Front Pediatr..

[4] Arendt F, Markiewitz A, Mestas M, Scherr S (2020). COVID-19 pandemic, government responses, and public mental health: Investigating consequences through crisis hotline calls in two countries. Soc Sci Med..

[5] Batchelor S, Stoyanov S, Pirkis J, Kõlves K (2021). Use of kids helpline by children and young people in Australia during the COVID-19 pandemic. J Adolesc Health..

[6] Liu S, Yang L, Zhang C, Xiang YT, Liu Z, Hu S (2020). Online mental health services in China during the COVID-19 outbreak. Lancet Psychiatry.

[7] Zhong B, Jiang Z, Xie W, Qin X (2020). Association of social media use with mental health conditions of nonpatients during the COVID-19 Outbreak: Insights from a national survey study. J Med Internet Res.

[8] Hill RM, Rufino K, Kurian S, Saxena J, Saxena K, Williams L (2021). Suicide ideation and attempts in a pediatric emergency department before and during COVID-19. Pediatrics..

[9] Isumi A, Doi S, Yamaoka Y, Takahashi K, Fujiwara T (2020). Do suicide rates in children and adolescents change during school closure in Japan? The acute effect of the first wave of COVID-19 pandemic on child and adolescent mental health. Child Abuse Negl..

[10] Sakamoto H, Ishikane M, Ghaznavi C, Ueda P (2021). Assessment of suicide in Japan during the COVID-19 pandemic vs previous years. JAMA Netw Open..

[11] Pirkis J, John A, Shin S, DelPozo-Banos M, Arya V, Analuisa-Aguilar P (2021). Suicide trends in the early months of the COVID-19 pandemic: an interrupted time-series analysis of preliminary data from 21 countries. Lancet Psychiatry.

[12] de Marques Miranda D, da Silva Athanasio B, Sena Oliveira AC, Simoes-e-Silva AC (2020). How is COVID-19 pandemic impacting mental health of children and adolescents?. Int J Disaster Risk Reduct.

[13] Colizzi M, Sironi E, Antonini F, Ciceri ML, Bovo C, Zoccante L (2020). Psychosocial and behavioral impact of COVID-19 in autism spectrum disorder: an online parent survey. Brain Sci..

[14] Breaux R, Dvorsky MR, Marsh NP, Green CD, Cash AR, Shroff DM (2021). Prospective impact of COVID-19 on mental health functioning in adolescents with and without ADHD: protective role of emotion regulation abilities. J Child Psychol Psychiatry..

[15] Sheridan DC, Cloutier R, Johnson K, Marshall R (2021). Where have all the emergency paediatric mental health patients gone during COVID-19?. Acta Paediatr.

[16] Leff RA, Setzer E, Cicero MX, Auerbach M (2021). Changes in pediatric emergency department visits for mental health during the COVID-19 pandemic: a cross-sectional study. Clin Child Psychol Psychiatry..

[17] Saban M, Myers V, Shachar T, Miron O, Wilf-Miron RR (2022). Effect of socioeconomic and ethnic characteristics on COVID-19 infection: the case of the ultra-orthodox and the Arab communities in Israel. J Racial Ethn Health Disparities..

[18] Kuznetsova A, Brockhoff PB, Christensen RHB (2017). lmerTest Package: tests in linear mixed effects models. J Statistical Software..

[19] Cost KT, Crosbie J, Anagnostou E, Birken CS, Charach A, Monga S (2022). Mostly worse, occasionally better: impact of COVID-19 pandemic on the mental health of Canadian children and adolescents. Eur Child Adolesc Psychiatry..

[20] de Díaz Neira M, Blasco-Fontecilla H, García Murillo L, Pérez-Balaguer A, Mallol L, Forti A (2021). Demand analysis of a psychiatric emergency room and an adolescent acute inpatient unit in the context of the COVID-19 pandemic in Madrid. Spain. Front Psychiatry..

[21] Bothara RK, Raina A, Carne B, Walls T, McCombie A, Ardagh MW (2021). Paediatric presentations to christchurch hospital emergency department during COVID-19 lockdown. J Paediatr Child Health..

[22] Kruizinga MD, Peeters D, van Veen M, van Houten M, Wieringa J, Noordzij JG, et al. The impact of lockdown on pediatric ED visits and hospital admissions during the COVID19 pandemic: a multicenter analysis and review of the literature. Eur J Pediatr. 2021;180:2271–9. 10.1007/s00431-021-04015-0 PMC7959585 33723971

[23] Delaroche AM, Rodean J, Aronson PL, Fleegler EW, Florin TA, Goyal M, et al. Pediatric Emergency Department Visits at US Children’s Hospitals During the COVID-19 Pandemic. Pediatrics. 147(4):e2020039628. 10.1542/peds.2020-039628 33361360

[24] Dror C, Hertz-Palmor N, Yadan-Barzilai Y, Saker T, Kritchmann-Lupo M, Bloch Y (2022). Increase in referrals of children and adolescents to the psychiatric emergency room is evident only in the second year of the COVID-19 pandemic&mdash;evaluating 9156 visits from 2010 through 2021 in a single psychiatric emergency room. Int J Environ Res Public Health.

[25] Ma Z, Idris S, Zhang Y, Zewen L, Wali A, Ji Y (2021). The impact of COVID-19 pandemic outbreak on education and mental health of Chinese children aged 7–15 years: an online survey. BMC Pediatr.

[26] Ertan D, El-Hage W, Thierrée S, Javelot H, Hingray C (2020). COVID-19: urgency for distancing from domestic violence. Eur J Psychotraumatol..

[27] Aolymat I (2021). A cross-sectional study of the impact of COVID-19 on domestic violence, menstruation, genital tract health, and contraception use among women in Jordan. Am J Trop Med Hyg.

[28] Myers K, Cain S (2008). Practice parameter for telepsychiatry with children and adolescents. J Am Acad Child Adolesc Psychiatry..

[29] Update C (2017). Telepsychiatry with children and adolescents. J Am Acad Child Adolesc Psychiatry.

[30] Butterfield A (2018). Telepsychiatric evaluation and consultation in emergency care settings. Child Adolesc Psychiatr Clin N Am.

[31] Revet A, Hebebrand J, Anagnostopoulos D, Kehoe LA, Gradl-Dietsch G, Anderluh M (2021). Perceived impact of the COVID-19 pandemic on child and adolescent psychiatric services after 1 year (February/March 2021): ESCAP CovCAP survey. Eur Child Adolesc Psychiatry.

[32] Reid S, Bhatt M, Zemek R, Tse S (2021). Virtual care in the pediatric emergency department: a new way of doing business?. CJEM.

[33] Zalsman G, Levy Y, Sommerfeld E, Segal A, Assa D, Ben-Dayan L (2021). Suicide-related calls to a national crisis chat hotline service during the COVID-19 pandemic and lockdown. J Psychiatr Res.

