# Correction: Pediatric psychiatric emergency rooms during COVID-19: a multi-center study

**DOI:** 10.1186/s12888-023-04561-x

**Published:** 2023-01-26

**Authors:** Galit Erez, Sol Yakubovich, Hadar Sadeh, Gal Shoval, Gila Schoen, Gal Meiri, Nimrod Hertz-Palmor, Tali Bretler, Yael Barzilai, Mariela Mosheva, Doron Gothelf, Yuval Bloch

**Affiliations:** 1 Shalvata Mental Health Center and Tel Aviv University, Hod Hasharon, Israel; 2 Shalvata Mental Health Center, Hod Hasharon, Israel; 3 Soroka Medical Center and Ben-Gurion University of the Negev, Be’er Sheva, Israel; 4 Geha Mental Health Center and Tel Aviv University, Petah Tikva, Israel; 5 Sheba Medical Center and Tel Aviv University, Ramat Gan, Israel; 6 Ziv Medical Center (Safed) and Bar-Ilan University, Safed, Israel

**Correction: BMC Psychiatry 22, 828 (2022)**

**https://doi.org/10.1186/s12888-022-04371-7**

Following publication of the original article [1], the authors identified an error in the author name of Tali Bretler.

The incorrect author name is: Tali Butler

The correct author name is: Tali Bretler

The author group has been updated above and the original article [1] has been corrected.
