# Supplementary material for: Pediatric psychiatric emergency rooms during COVID-19: a multi-center study
Source: BMC Psychiatry. 2022 Dec 27;22:828. doi: 10.1186/s12888-022-04371-7 (PMC9793352; doi:10.1186/s12888-022-04371-7)
Supplement: Supplementary file 1 — Additional file 1. [file 12888_2022_4371_MOESM1_ESM.docx]

**Additional file 1**

**Classification of diagnoses, by ICD-10 code***

| Category | Code | Name |
| --- | --- | --- |
| Stress-related, anxiety, and mood disorders | F43 | Reaction to severe stress, and adjustment disorders |
|  | V628 | Adjustment |
|  | F40 | Phobic anxiety disorders |
|  | F41 | Other anxiety disorders |
|  | F44 | Dissociative [conversion] disorders |
|  | F45 | Somatoform disorders |
|  | F940 | Elective mutism |
|  | F93 | Separation anxiety disorder of childhood |
|  | 3000 | Anxiety |
|  | F32 | Depressive episode |
|  | F33 | Recurrent depressive disorder |
|  | F34 | Persistent mood [affective] disorders |
|  | F38 | Other mood [affective] disorders |
|  | F39 | Unspecified mood [affective] disorder |
| ADHD, conduct, and learning disabilities | F90 | Hyperkinetic disorders |
|  | 3140 | ADHD |
|  | F91 | Conduct disorders |
|  | F92 | Mixed disorders of conduct and emotions |
|  | F80 | Specific developmental disorders of speech and language |
|  | F81 | Specific developmental disorders of scholastic skills |
|  | F83 | Mixed specific developmental disorders |
| Severe Mental Disorders | F50 | Eating disorders |
|  | 299 | Autism |
|  | F941 | Reactive attachment disorder of childhood |
|  | F84 | Pervasive developmental disorders |
|  | F42 | Obsessive-compulsive disorder |
|  | F95 | Tic disorders |
|  | F55 | Abuse of non-dependence-producing substances |
|  | F98 | Other behavioural and emotional disorders with onset usually occurring in childhood and adolescence |
|  | F070 | Organic personality disorder |
|  | F70 | Mild mental retardation |
|  | F71 | Moderate mental retardation |
|  | F72 | Severe mental retardation |
|  | F952 | Combined vocal and multiple motor tic disorder [de la Tourette] |
|  | F20-F29 | Schizophrenia, schizotypal and delusional disorders |
|  | F30 | Manic episode |
|  | F31 | Bipolar disorder |
|  | F6 | Disorders of adult personality and behavior |
|  | F070 | Klüver–Bucy syndrome |
|  | F48 | Neurasthenia |
|  | F1 | Multi-infarct dementia |
|  | P721 | Transitory neonatal hyperthyroidism |
|  | F78 | Other mental retardation |
|  | F79 | Unspecified mental retardation |
|  | F002 | Dementia in Alzheimer disease, atypical or mixed type |
|  | 4380 | Intellectual disability |
|  | F76 | Malingerer |
|  | F51 | Nonorganic sleep disorders |

* Sub-codes were also included.
